# Supplementary material for: The shape of cancer relapse: Topological data analysis predicts recurrence in paediatric acute lymphoblastic leukaemia
Source: PLoS Comput Biol. 2023 Aug 14;19(8):e1011329. doi: 10.1371/journal.pcbi.1011329 (PMC10468039; doi:10.1371/journal.pcbi.1011329)
Supplement: Attachment — Submitted filename: Response to reviewers.pdf [file pcbi.1011329.s006.pdf]

Oxford Mathematics

Andrew Wiles Building
Radcliffe Observatory Quarter
Woodstock Road.
Oxford OX2 6GG
United Kingdom

+44 (0)1865 615149
helen.byrne@maths.ox.ac.uk
www.maths.ox.ac.uk

UNIVERSITY OF OXFORD
Mathematical Institute

Feilim Mac Gabhann & Jason Papin
Editors-in-chief
PLoS Computational Biology

Dear Editors,

We thank the referees for the time and effort that they invested in reviewing our manuscript titled "*The shape of cancer relapse: topological data analysis predicts recurrence in paediatric acute lymphoblastic leukaemia*." We would like to express our gratitude for their valuable feedback and helpful suggestions.

We have carefully revised our manuscript in response to the reviewers' comments and addressed some technical requirements indicated by the PLoS Computational Biology office. We detail below our responses to the points that they raised together with the corresponding changes to our article.

We hope that our updated manuscript addresses the reviewers' comments, and we thank them once again for their constructive criticism, which has undoubtedly helped us to improve the quality of our work.

We look forward to hearing from you soon.

On behalf of all authors and with kind regards,

Corresponding author signature

Reviewer 1

1. *On page 8, the analysis for the 4-dimensional point clouds (before the discussion of persistence images) could be better motivated. Is the point of using the FlowSom algorithm simply to confirm the 0-dimensional PH results?*

   Our aim in clustering with FlowSom was indeed to confirm the 0-dimensional PH results. To clarify this, we have included additional statements in the Results section (see lines 158 and 200) that clearly outline our goal of computing differences between patients based on the number of clusters, which can be achieved using either FlowSom or TDA.

2. *Is there any insight gained from the fact that there are significant differences between the discovery and validation sets for the 4-dimensional analysis of biomarkers? Since there are no differences based on the whole dataset, it seems like this would not be important to report on (say, in Supplement 2.3a).*

   This is indeed a good point, given that the primary objective of undertaking the 4-dimensional analysis was to leverage the multidimensional nature of the data. However, despite the comprehensive analysis of the whole dataset, including both discovery and validation sets, we detected no significant differences. As a result, we have simplified this statement in the main text (line 193), while retaining it in the supplementary section for those readers who may be interested in exploring this aspect in greater detail.

3. *What is the motivation for performing classification for the low or intermediate risk patients separately?*

   We performed classification for the low-intermediate risk patients separately due to clinical interest. It would be advantageous to identify those patients whose initial risk assessment was incorrect and then to reclassify them. As shown in Table S2, it is noteworthy that 85.7% of all patients who experience relapse were initially categorized as having a low-intermediate risk profile. We have highlighted this in the main text, motivating this classification (see line 251).

4. *Choosing thresholds for persistence threshold curves in applications is always a challenge. It would be interesting if the authors commented on any insights gained on the appropriate choices for flow cytometry applications.*

   We appreciate the reviewer's comment, as this was the main reason that for constructing the persistence threshold (PT) curves. We performed a parameter scan across a range of thresholds, and the selection of these intervals (see lines 513-516 in Methods) was based on the finding of significant differences between the NR and R patients' PT curves. While we did not find a consistently optimal threshold or range, there is an indication that, for this type of flow cytometry data, dimension 1 features tend to present significant differences at lower thresholds than dimension 0 features. This trend is not strong enough to give a general recommendation, so we suggest that a similar parameter scan be performed when analysing new data, even if these are FC data. We have clarified this point in the main text (line 322).

5. *More generally, the discussion would be improved by a discussion of parameter choices (grid, coefficients, etc) for both PH (say, for the PI images) and for the ML methods used, such as SVM. In particular, does this work suggest ways to choose these parameters in an informed way?*

   We agree that for both classification and PI construction the choice of parameter values is very important. We have now included a brief discussion to clarify this: First, we emphasise that the selection of Gaussian spreads and pixel grids was carried out in Figure

S21, where we identified parameter values that yielded significant differences between NR and R patients. Secondly, as outlined in the 'Methods' section, we employed a randomized approach to select SVM parameters (line 546). This approach involved splitting the training set and performing internal cross-validations across a logarithmic range of values for the parameters $C \in [10^{-2}, 10^{13}]$ and $\gamma \in [10^{-9}, 10^{3}]$. Finally, given the variability of the optimal hyperparameters C and γ found in Table S2, S3 and S4, we performed a full analysis for C=10 and $\gamma=10^{-3}$, and include the associated results in Tables S6, S7 and S8. We have clarified our approach in the captions of Tables 1 and 2. We have also included a brief summary of the choice of parameter values in the discussion (see lines 330-336).

Reviewer 2

1. *The following statement presents an exceptionally narrow and outdated view of ML: "Data analysis methods, such as principal component analysis or machine learning (ML) methods (neural networks, support vector machines, etc) could also be used for classification. However, these methods focus on identifying linear relationships between the biomarkers and do not characterise the shape of the data. Further, the results are often difficult to interpret."*

   Several methods for non-linear dimensionality reduction, manifold and representation learning are available for the analysis of biomedical data. Furthermore, there are multiple model-agnostic techniques for performing feature selection and interpretation at both global and local levels. Please see "Interpretable Machine Learning" by Christoph Molnar (available online) for more information.

   Thank you for your comments. We have removed our original statement regarding the linearity and interpretability of Machine Learning methods. In addition, we have reinforced the idea that combining these methods with Topological Data Analysis (TDA) can provide a means of including shape information as markers, thereby enhancing their value. We have revised our motivation for integrating TDA and Machine Learning in the Introduction (lines 47-58) and commented on the relevance of interpretability of biomedical data and machine learning (new Ref. https://doi.org/10.1016/j.asoc.2021.108391, lines 95-97).

2. *How does the TDA-based approach compare against other supervised and unsupervised methods for patient classification, e.g. CellCnn (Arvaniti and Claassen, Nature Communications, 2017), Citrus (Bruggner et al, PNAS, 2014), FloReMi (Van Gassen et al, Cytometry A, 2016)?*

   We thank the reviewer for bringing these references to our attention. Most of the machine learning (ML) techniques that have been applied to FC data (as discussed in the review from new Ref. https://www.frontiersin.org/articles/10.3389/fimmu.2021.787574/full), concentrate on dimensionality reduction and cell population identification. CellCNN, CITRUS, and FloReMi employ convolutional neural networks (CNN), a LASSO model, and a Random Forest (RF) model, respectively, to perform classification. All of these approaches, however, have one thing in common: they rely on the expression level of cell markers, i.e., their fluorescence intensity values, for classification and feature extraction. However, the approach presented in this paper focuses on complementary attributes: the shape of the data-point cloud, which is described and quantified by means of the TDA. This approach is also inspired by the procedure used to analyse flow cytometry (FC) in clinical practice, which is based on manual assessment of the shapes of the projected biomarkers (lines 36-41). We have emphasized this point in the introduction (lines 47-58).

3. *Is it possible to achieve similar or better classification accuracy with lower computational cost by training the classifier directly on 2D projection coordinates (e.g. using kernel density estimation) or other low dimensional embeddings obtained using an autoencoder, Isomap, MDS, etc.?*

   As mentioned in our response to point 2 above, we understand that there are specific tools for dimensionality reduction in FC. However, the 2D representations obtained through these dimensionality reduction tools are still be based on the values of fluorescence intensity. By contrast, persistence images provide a means to generalize results derived from the shape of the data-point cloud. In our analysis, we seek to exploit particular aspects of the multiscale TDA descriptors, by working with projections of the FC data that are usually used in the clinic.

4. *Dimension 0 homology is sensitive to total number of points in the point cloud. Were equal number of samples used to compute PH for each patient? Were the PT curves and persistence images normalized?*

   This is indeed a problem when performing dimension 0 homology. We applied a number of normalization processes to the data to avoid this effect. First, we reduced the number of samples to one for all patients using k-nearest neighbor imputation (line 464). Next, we applied a rescaling transformation to normalize each patient sample and reduce batch effects (line 466). Additionally, we reduced the data to a fixed number of landmarks, as stated in response to question #8 (lines 474-477). We further normalized the PT curves to the number of bars and % of bars, as illustrated in Figs 5. and S10-S15, and mention this in the TDA and PI section (line 511). Finally, all PI were mapped onto squared pixeled matrices of a fixed length, as stated in line 520.

5. *The dataset consisted of 16 R (17%) and 80 NR (83%) patients. These are divided into discovery and validation groups. The discovery group (Datasets 1 and 2, Table S1) contains approx. 25% R and 75% NR patients from two hospitals, while the validation group (Dataset 3, Table S1) contains 7% R and 93% NR patients from another hospital. Why were the training and validation groups generated in this manner? Would a naive classifier that always predicts NR achieve 0.93 accuracy? Why not merge data from different hospitals and use stratified k-fold validation?*

   Thank you for your comment. In response, we have now fixed the hyperparameters C and γ, and performed stratified k-fold cross-validation on all datasets, both with and without uppersampling, as detailed in Table 2. To provide a concise summary of our results without overwhelming the reader, we have included new Tables S6, S7 and S8 in the Supplementary Section, while Tables 1 and 2 serve as succinct summaries of our findings. We have clarified our approach in the captions of Tables 1 and 2.

6. *Is the accuracy of model predictions in Table 1 the mean values across 6 folds? Please include std. dev., precision, recall and F1 scores in your results. For unbalanced data, precision, recall and PRC curves are more informative than accuracy and AUC scores.*

   As stated in our response to the previous comment, we have included this information in the new Tables S6, S7 and S8.

7. *'Robustness to noise' is cited as an inherent advantage of topological methods. While the stability theorem guarantees robustness to small perturbations in the point cloud, TDA is not robust to outliers. Outlier removal in data preprocessing is important in this regard. Please consider highlighting this in relevant methods section.*

   We agree that outliers can introduce spurious topological features. We therefore preprocessed our data specifically to avoid outliers. We have now revised the 'Methods' section, to clarify the procedure we used to ensure outlier removal (see line 467).

8. *Each patient was subset to 10^5 cells. From these, 10^4 landmarks were selected. For dim 2 persistent homology, the number of landmarks was further reduced to 10^3. Did the authors consider using Ripser++ or weak alpha filtrations to speed up the computation?*

   We thank the reviewer for this helpful suggestion, as computational cost is a drawback when performing TDA analyses and we will certainly bear it in mind for future projects. When we began our work, in early 2020, we did not consider using either of these methods and were indeed unaware of the existence of Ripser++. While using weak alpha complexes would certainly speed up our computations, we note that the Giotto-TDA documentation specifically warns about problems with interpretation (see

https://giotto-ai.github.io/gtda-docs/0.3.0/modules/generated/homology/gtda.homology.WeakAlphaPersistence.html). Interpretation of topological features is very important to us as we are working closely with clinicians. We therefore prioritise methods with intuitive interpretation over computational speed. We have now, however, highlighted the potential of Ripser++ to perform the analyses (see line 561).

#Reviewer 3

1. *The authors used an oversampling method to balance the data when they want to perform classification using Logistic Regression and Support Vector Machines. However, when using Random Forests to choose the pairwise biomarkers, they used the original data set without oversampling. Could this create biased results in choosing the pairwise biomarkers?*

   We agree that this could have biased the results when choosing the pairwise biomarkers. When we performed the same Random Forest analyses using oversampling, there were no significant differences in the AUCs between different biomarkers. Our decision to use the results without oversampling was based on this observation, together with the fact that combinations using markers CD10-CD20-CD38-CD45 are biologically reasonable, given their interpretability regarding bad/good prognosis and subpopulation distinction (see lines 148-152). These Random Forest analyses, which use oversampling and stratified k-fold, are included in a new Dataset S1 and discussed in the supplementary (lines 149-150) and main text (lines 142-144).

2. *On lines 488-489 the authors mention that they used a Random Forest algorithm due to its accuracy and interpretability, and then on lines 500-501 the authors mention that they used SVMs since they provide good decision surfaces. Can the authors comment on why they expect different results from a Random Forest model as opposed to an SVM? One would expect a Random Forest to perform similar to an SVM since it relies on bootstrap aggregation method, which has the consequence of smoothing the decision surface. (See Breiman 1996, Bagging Predictors, Machine learning 24, 123-140).*

   We thank the reviewer for posing this question. In response, we did not favor one classifier over another; rather, we regarded both as equally valid, as the reviewer suggests. We employ both because we perform two distinct classification tasks. The first task involves determining the most important pairwise combination of markers, for which we use a Random Forest classifier. The second task involves using PIs to classify relapsing patients, which we achieve via an SVM. We have now clarified our approach within the Machine Learning Section (line 525) of the Methods section.

Technical Requests

1. New funding information was updated (see lines 679-681; 687-689).
2. Data availability was amended. Flow cytometry data was uploaded to a flow repository (see lines 572-573).
3. SI Legends were included not only in SI but in the main manuscript (see lines 600-652).
4. Name of participating local institutions was included, as well as indications on formal consent and anonymity (see lines 436-443).
5. Possibly copyrighted material (icons in Figures) were changed in Figures 1-5 and S9, using icons from www.openclipart.org and www.bioicons.com, all with License CC0 (open release, no need to cite authors).
