# Supplementary material for: The shape of cancer relapse: Topological data analysis predicts recurrence in paediatric acute lymphoblastic leukaemia
Source: PLoS Comput Biol. 2023 Aug 14;19(8):e1011329. doi: 10.1371/journal.pcbi.1011329 (PMC10468039; doi:10.1371/journal.pcbi.1011329)
Supplement: Attachment — Submitted filename: 2nd_revision.pdf [file pcbi.1011329.s007.pdf]

Oxford Mathematics

Andrew Wiles Building
Radcliffe Observatory Quarter
Woodstock Road.
Oxford OX2 6GG
United Kingdom

+44 (0)1865 615149
helen.byrne@maths.ox.ac.uk
www.maths.ox.ac.uk

UNIVERSITY OF OXFORD
Mathematical Institute

Feilim Mac Gabhann & Jason Papin
Editors-in-chief
PLoS Computational Biology

27 June 2023

Dear Editors,

We thank the referees for the time and effort that they invested in reviewing our manuscript titled "*The shape of cancer relapse: topological data analysis predicts recurrence in paediatric acute lymphoblastic leukaemia*." We would like to express our gratitude for their positive feedback and helpful suggestions.

We have carefully revised our manuscript in response to the comments from Reviewer 2. We detail below our responses to the points that they raised and the corresponding changes.

We trust that our updated manuscript addresses the reviewer's comments, and we thank them once again for their constructive feedback, which has undoubtedly had a positive impact on our work. We look forward to hearing from you soon.

On behalf of all authors and with kind regards,

Corresponding author signature

Reviewer 2

1. *Please specify the numbers of R/NR samples in Fig. S8(A) and in Tables S6-S8. For example, in Table S6, approx. 2 NR samples are being classified in each fold. The total number of samples is 77 (why?). Each fold will contain approx. (1/6)*77*0.1667 = 2 NR samples (80 R + 16 NR => 16.67% NR samples in each of the 6 stratified folds).*

We have now included the number of R and NR samples per fold in Fig. S8(A) and Tables S6-S8. We have also updated the information in Tables 2 and S6-S8, so that they now include the full dataset that we studied (i.e., 80 R + 16 NR samples). Additionally, we have included a new Table S9 which includes the classification of patients with low-intermediate risk but performing oversampling during training.

2. *Correct the typo in Table S6 (precision = 75.00)*

Thank you, we have corrected this typo and updated Tables S6-S8 (see previous response).

3. *Update and refactor your GitHub code (see comments pertaining to PLOS Data policy)*
   a. *The code is provided with no documentation or comments. Basic code formatting guidelines are not followed.*
   b. *The code does not work. For example, in Result1/__Step 01.py the line tube=flow.Tube(file=datafile) returns an error because flow was never defined or imported.*
   c. *The repository was last updated 2 years ago (on Dec 21, 2021). The code is outdated and does not reproduce any of the revised results (precision, recall, etc. in Tables S6-S8)*

We thank the reviewer for bringing these points to our attention. We have expanded the documentation so that clear instructions about the inputs and outputs needed for each piece of code are indicated in the Readme file on Github (https://github.com/salvadorchulian/shapecancerrelapse). We have also ensured the independence of each computational step, so that interested readers can select the data they would like to analyse and use only the relevant .py file. We have updated the repository to include the revised results from Tables S6-S8.

4. *I would prefer to see at least one comparison with a non-TDA technique. You can classify the 2D projections used in clinical practice (no dimensionality reduction) by directly training on the coordinates instead of the PH features. You could also consider training your classifier on other shape features (elongation, convexity, etc.) to show that TDA perform equally well if not better.*

We appreciate the reviewer's comment. We performed a non-TDA classification, using statistical analysis, in a previous paper (https://doi.org/10.3390/cancers13010017). In this study, we used the data from Hospitals 1 and 2, and achieved an accuracy of 80% using individual clinical parameters obtained from flow cytometry data (e.g. CD38). We have now highlighted these results in the discussion (lines 349-356). In this new paragraph, we also remind the reader of the 86% accuracy obtained from the Random Forest classification performed on the clinical variables, (see line 235) and the application of the FlowSom Algorithm to compute the number of clusters in the two-dimensional CD10-CD20 projection, where we obtained marginally significant results (p=0.18, see line 207). All accuracies calculated using non-TDA methods are smaller than those obtained using TDA.
